# Case Definition of Chronic Pulmonary Aspergillosis in Resource-Constrained Settings

**DOI:** 10.3201/eid2408.171312

**Published:** 2018-08

**Authors:** David W. Denning, Iain D. Page, Jeremiah Chakaya, Kauser Jabeen, Cecilia M. Jude, Muriel Cornet, Ana Alastruey-Izquierdo, Felix Bongomin, Paul Bowyer, Arunaloke Chakrabarti, Sara Gago, John Guto, Bruno Hochhegger, Martin Hoenigl, Muhammad Irfan, Nicholas Irurhe, Koichi Izumikawa, Bruce Kirenga, Veronica Manduku, Samihah Moazam, Rita O. Oladele, Malcolm D. Richardson, Juan Luis Rodriguez Tudela, Anna Rozaliyani, Helmut J.F. Salzer, Richard Sawyer, Nasilele F. Simukulwa, Alena Skrahina, Charlotte Sriruttan, Findra Setianingrum, Bayu A.P. Wilopo, Donald C. Cole, Haileyesus Getahun

**Affiliations:** University of Manchester, Manchester, UK (D.W. Denning, I.D. Page, F. Bongomin, P. Bowyer, S. Gago, R.O. Oladele, C. Sriruttan, F. Setianingrum, B.A.P. Wilopo);; Wythenshawe Hospital Manchester University NHS Foundation Trust, Manchester (D.W. Denning, I.D. Page, S. Moazam, M.D. Richardson, R. Sawyer);; The Global Action Fund for Fungal Infections, Geneva, Switzerland (D.W. Denning, J. Guto, J.L. Rodriguez Tudela);; Kenya Medical Research Institute, Nairobi, Kenya (J. Chakaya, V. Manduku);; Aga Khan University, Karachi, Pakistan (K. Jabeen, M. Irfan);; Olive View–UCLA Medical Center, Sylmar, California, USA (C.M. Jude);; Centre Hospitalier Universitaire Grenoble Alpes, Grenoble, France (M. Cornet);; Instituto de Salud Carlos III, Madrid, Spain (A. Alastruey-Izquierdo);; Postgraduate Institute of Medical Education and Research, Chandigarh, India (A. Chakrabarti);; Federal University of Health Sciences of Porto Alegre, Porto Alegre, Brazil (B. Hochhegger);; University of California San Diego, San Diego, California, USA (M. Hoenigl);; Medical University of Graz, Graz, Austria (M. Hoenigl);; Center for Biomarker Research in Medicine, Graz (M. Hoenigl);; Lagos University Teaching Hospital, Lagos, Nigeria (N. Irurhe);; Nagasaki University Graduate School of Biomedical Sciences, Nagasaki, Japan (K. Izumikawa);; Mulago Hospital and Makerere University, Kampala, Uganda (B. Kirenga); University of Lagos, Lagos (R.O. Oladele);; Universitas Indonesia, Jakarta, Indonesia (A. Rozaliyani, F. Setianingrum);; Research Center Borstel, Borstel, Germany (H.J.F. Salzer);; Royal Liverpool University Hospital, Liverpool, UK (N.F. Simukulwa);; The Republican Scientific and Practical Centre for Pulmonology and TB, Minsk, Belarus (A. Skrahina, C. Sriruttan);; National Institute for Communicable Diseases, Johannesburg, South Africa (C. Sriruttan);; University of the Witwatersrand, Johannesburg (C. Sriruttan);; Universitas Padjadjaran, Bandung, Indonesia (B.A.P. Wilopo);; University of Toronto, Toronto, Ontario, Canada (D.C. Cole); World Health Organization, Geneva (H. Getahun)

**Keywords:** Tuberculosis, Aspergillus, antibody, aspergilloma, imaging, developing countries, resource-constrained settings, tuberculosis and other mycobacteria, fungi

## Abstract

Chronic pulmonary aspergillosis (CPA) is a recognized complication of pulmonary tuberculosis (TB). In 2015, the World Health Organization reported 2.2 million new cases of nonbacteriologically confirmed pulmonary TB; some of these patients probably had undiagnosed CPA. In October 2016, the Global Action Fund for Fungal Infections convened an international expert panel to develop a case definition of CPA for resource-constrained settings. This panel defined CPA as illness for >3 months and all of the following: 1) weight loss, persistent cough, and/or hemoptysis; 2) chest images showing progressive cavitary infiltrates and/or a fungal ball and/or pericavitary fibrosis or infiltrates or pleural thickening; and 3) a positive *Aspergillus* IgG assay result or other evidence of *Aspergillus* infection. The proposed definition will facilitate advancements in research, practice, and policy in lower- and middle-income countries as well as in resource-constrained settings.

The differential diagnosis for pulmonary tuberculosis (TB) is wide and includes nontuberculous mycobacteria (NTM) infection, endemic fungal infections such as coccidioidomycosis and histoplasmosis, allergic bronchopulmonary aspergillosis, and chronic pulmonary aspergillosis (CPA) (*1**–**7*). Sequelae of pulmonary TB, such as bronchiectasis and restricted lung capacity, can mimic infection relapse (*8**–**10*). Accurate diagnosis is essential for adequate treatment.

The 2015 World Health Organization annual report notes that ≈2.2 million (≈43%) of 5.2 million cases of incident pulmonary TB were clinically diagnosed or smear-negative (*11*). Only 21%–40% of smear-negative pulmonary TB cases are culture positive (*12**,**13*). Exclusion of alternatives is challenging in many lower- and middle-income countries (*14*). The World Health Organization report comments, “Most clinical features of TB and abnormalities on X-ray or histology results generally associated with TB have low specificity, which may lead to false diagnoses of TB, and hence to people being enrolled on TB treatment unnecessarily” (*11*).

Although coccidioidomycosis, histoplasmosis, and paracoccidioidomycosis are regionally confined, aspergillosis is global. Each year, an estimated 373,000 new CPA cases complicate treated pulmonary TB within 12 months of completion of anti-TB therapy; the 5-year period prevalence is 1,174,000 (range 397,000–2,088,000) cases (*9*). This wide range results from several factors, notably the extrapolation of CPA diagnosis from a limited UK dataset of only 544 patients with pulmonary cavities (*15*), substantial variability in the published frequency of cavitation after treatment of pulmonary TB, absence of an estimate of CPA prevalence among patients without cavities, and lack of knowledge of the effects of concurrent HIV infection. The incidence and prevalence of CPA are not known but are probably underestimated, in part because CPA occurs in patients with active pulmonary TB, as a sequela of prior pulmonary TB (*16*), or as a complication of other pulmonary disorders with symptoms similar to those of pulmonary TB, and is incorrectly diagnosed and treated as pulmonary TB (*17*).

Diagnostic guidelines for CPA have recently been published in English and Japanese, emphasizing the central role of advanced imaging and serologic testing for *Aspergillus* (*18**–**20*). Unfortunately, these diagnostics are infrequently available in many resource-constrained settings. In October 2016, the Global Action Fund for Fungal Infections convened an international panel to develop an operational definition of CPA for research and clinical care in resource-constrained settings. The panel’s goals were to adapt the existing European Society for Clinical Microbiology and Infectious Diseases and European Respiratory Society (*19*) and Infectious Diseases Society of America guideline case definitions of CPA (*18*) to promote research so that critical data will be available to inform policy and practice, including surveillance, and to enable individualized clinical care for optimal patient management.

## Methods

### Literature and Existing Guidelines

We built on the work of 2 recent CPA expert panels (*18**–**20*). These panels undertook comprehensive searching, appraisal, and synthesis of the relevant literature, including diagnosis and case definitions. We included these papers in a package of materials relevant to diagnosis in different clinical contexts (i.e., underlying disease in patients in whom CPA is developing, CPA and clinically diagnosed pulmonary TB, radiologic assessment and characteristics of CPA, and comparisons of laboratory diagnosis with different immunoassays).

### Workshop Participants

The Global Action Fund for Fungal Infections (https://www.gaffi.org) invited 36 experts from all regions of the world, according to expertise. The experts had already implemented CPA diagnostic capacity or were in the process of doing so. Participants also included experts from lower- and middle-income countries that had active clinical and public health programs focused on respiratory diseases including TB. Clinical expertise included internal medicine, pulmonary disease, infectious disease, critical care, thoracic radiology, medical microbiology, and medical mycology, as well as various health system organizational roles and levels (e.g., secondary care consultants, national reference laboratories, national research centers, and international health organizations). Of the 36 invited experts, 33 (the authors) attended a workshop in Liverpool, UK.

### Operational Definitions Indicator Selection

Morning presentations and discussions built on prior reading material and provided all participants evidence of CPA burden, risk factors, clinical presentations, diagnostic tools, treatment options, recurrences, and prior case definitions. Three facilitated afternoon breakout groups started with CPA diagnostic indicator types (i.e., clinical presentation, radiology, medical microbiology–mycology–immunology) and discussed options for diagnosis, focusing on secondary care levels and above for lower- and middle-income countries. A recorder took notes, and the pros and cons of different indicators were shared in a plenary session. Subsequently, 3 cross-indicator groups worked to bring the indicators together, constructing key criteria for CPA with different clinical or radiologic presentations.

### Development of Case Definitions 

Plenary discussion participants compared and contrasted the different approaches and moved toward operational definitions. On the basis of consolidated notes from breakout groups and plenary sessions, descriptions of possible, probable, and confirmed CPA were synthesized to garner consensus on the most critical elements of the diagnosis. Simple graphic representations were developed, shared with breakout group leaders for feedback, and subsequently revised through iteration. Further iteration on the key elements of the algorithms to be used in the field and minimal definitional requirements were conducted via email and online file-sharing services, simplifying the definition to a single, composite definition and algorithm.

## Results

Modern diagnostic criteria for CPA date from 2003 (*21*) and have been used in some prospective clinical trials (Table 1) and refined for specific purposes. The consensus group considered diagnostic criteria in 3 sections: clinical features, radiologic criteria, and microbiological criteria.

**Table 1:** Published diagnostic features and criteria for chronic pulmonary aspergillosis*

Parameter	Reference					
	(*21*)	(*22*)	(*23*)	(*24*)†	(*25*)	(*26*)
Symptoms	>1 of the following for 3 mo: WL, productive cough, hemoptysis plus absence of overt immunosuppression	>1 of the following (no duration specified): fever, WL, sputum production, cough, hemoptysis, fatigue, shortness of breath	Performance status 1–2	All of the following required for 1–6 mo: fever, cough, sputum production, weight loss	>1 of the following for 3 mo: weight loss, productive cough, hemoptysis plus absence of overt immunosuppression	“Significant pulmonary and/or systemic symptoms for 3 months or more”; no specific symptoms listed
Radiology	>1 of the following: cavitary lesion with paracavitary fibrosis, new or expanding cavity on serial imaging	>1 of the following: new infiltrates, cavity formation, expansion of preexisting cavities; with or without the following: pericavitary infiltrates, adjacent pleural thickening	Compatible chest CT scan or photo-graphically confirmed endoscopic lesion	Cavitary pulmonary lesion with evidence of pericavitary infiltrates and adjacent pleural thickening with/without fungal ball	>1 of the following: cavitary lesion with paracavitary fibrosis, new or expanding cavity on serial imaging	Both required: >1 pulmonary cavities with either thick or thin wall, possibly containing aspergilloma or irregular intraluminal material; overt radiologic progression over >3 mo required (new cavities, increasing pericavitary infiltrates, or increasing fibrosis)
*Aspergillus* antibody/ culture	Either positive precipitins, or, culture from pulmonary or pleural cavity	>1 of the following: platelia serum galactomannan index >1.0, positive precipitins, positive (1,3)-β-D-glucan, evidence of *Aspergillus* spp. by molecular diagnosis, culture or pathological findings	Positive serologic test required by both of the following: precipitins by CIE with >2 lines, second serologic test positive by any method; and microbiological evidence by 1 of the following sources from BAL or sputum samples: >2 or more positive cultures, 1 positive culture and positive microscopy	Culture from sputum or BAL mandatory, antibodies not required	Either raised *Aspergillus*-specific IgG or culture from pulmonary or pleural cavity	If fungal ball present: *Aspergillus*-IgG/precipitins or other evidence of *Aspergillus*. If no fungal ball but >1 cavities, then any of the following: *Aspergillus-*specific IgG, *Aspergillus* precipitins, strongly positive *Aspergillus* antigen or DNA in respiratory fluids, percutaneous or excision biopsy showing fungal hyphae on microscopy, growing *Aspergillus* from a cavity. These tests on respiratory samples not sufficient in isolation: culture, PCR, microscopy
Inflammatory markers	Raised levels of either: CRP, ESR, plasma viscosity	>1 of the following raised: leukocyte count, CRP, ESR	Not required	Not required	Raised levels of either: CRP, ESR	Not required
Exclusion of other pathogens	Required with the following examples: mycobacteria, endemic mycoses	Lack of improvement with >3 d of broad-spectrum antimicrobial drugs required; patients with infectious diseases other than aspergillosis excluded	Not required	Required with the following examples: TB, other mycoses, granulomatosis with polyangiitis, ABPA, invasive aspergillosis, simple aspergilloma†	Not specifically required	Required with the following examples: TB, atypical mycobacteria, necrotizing lung cancer, pulmonary infarction, vasculitides, rheumatoid nodule, histoplasmosis/ coccidioidomycosis/ paracoccidioido-mycosis in those with relevant travel history

*ABPA, allergic bronchopulmonary aspergillosis; BAL, bronchoalveolar lavage; CIE, counterimmunoelectrophoresis; CNPA, chronic necrotizing pulmonary aspergillosis; CRP, C-reactive protein: CT, computed tomography; ESR, erythrocyte sedimentation rate; TB, tuberculosis; WL, weight loss. †This definition includes CNPA cases with 1–3 mo symptoms. Note: precipitins sensitivity even lower than culture. Pleural thickening seems to be a mandatory criterion.

### Clinical Features

#### Underlying Diseases

Most CPA patients have prior or concurrent underlying pulmonary disease. Possible risk factors for development of CPA are cavities in the lung caused by pulmonary TB, sarcoidosis, previous *Pneumocystis* pneumonia, bullae or lung cysts, lung abscess, pulmonary infarction, pulmonary fibrosis, healed abscess cavities, cavitary bronchogenic carcinoma, and infection by NTM (*21**,**26**–**30*). In many countries, pulmonary TB is the most common disorder that precedes CPA (*9*). The most common differential diagnosis is pulmonary mycobacterial infection.

#### Duration

The duration of disease required to define CPA used by previous studies has been 1–6 months, usually 3 months. (Table 1). The expert panel consensus was to endorse a 3-month duration as a criterion for diagnosis of CPA. In patients with preexisting pulmonary disease, who often have chronic signs and symptoms, a change in pattern or severity of clinical presentation is considered the trigger point for the 3-month duration. In patients with few or unchanged signs and symptoms, documentation of 3-month duration may also be confirmed with radiographic findings of progression of cavitation, pericavitary infiltrates or fibrosis, development of a fungal ball (which takes weeks to form), or microbiological data.

#### Signs and Symptoms

Most patients with CPA experience clinical signs and symptoms, although some are asymptomatic and show progression radiologically only. The most distinctive and alarming sign is hemoptysis/hemosputum. Hemoptysis develops in ≈12%–43% of CPA patients (*23**,**25**,**31**,**32*) and varies from blood streaking in sputum to massive and fatal hemoptysis. Hemoptysis may occur in patients with TB, but it is only streaking of sputum with blood and usually not severe. Another characteristic symptom is mild but persistent chest pain, discomfort, or tightness, experienced by up to 37% of patients. Weight loss and fatigue are also common, although not universal. Cough (usually productive) and dyspnea are common but not sufficiently distinctive to distinguish CPA from other pulmonary disorders, including pulmonary TB. Fever or pyrexia is uncommon in CPA patients and, if present, may indicate a concurrent or alternative diagnosis or subacute invasive aspergillosis. Night or day sweats are occasionally reported but are not discriminatory.

The consensus of the panel was that the diagnosis of CPA required the presence of >1 symptoms persisting for 3 months and radiologic progression, a scenario present in most cases. Two other clinical scenarios that also qualify for the diagnosis of CPA are radiologic evidence of a simple aspergilloma (with or without symptoms) and characteristic radiologic appearance without symptoms but showing definite radiologic progression.

### Radiologic Criteria

The focus of the discussion revolved around use of chest radiographs alone to diagnose CPA; requiring computed tomography (CT), which is often unavailable, might delay diagnosis. Multiple studies have demonstrated that CT is more sensitive than chest radiography for demonstrating several features, namely, >1 fungal balls, pulmonary nodules, multiple cavities, and disease in the apices and retrocardiac space (*33*). Considering the limited availability of CT in many healthcare settings, the radiologic criteria adopted are based solely on chest radiograph findings. Where available, CT is recommended in the context of clinical and microbiological suspicion of CPA with a nondiagnostic chest radiograph.

#### Fungal Ball or Aspergilloma, Intracavitary Material, or Fluid Level on Chest Radiograph

**Figure 1:**
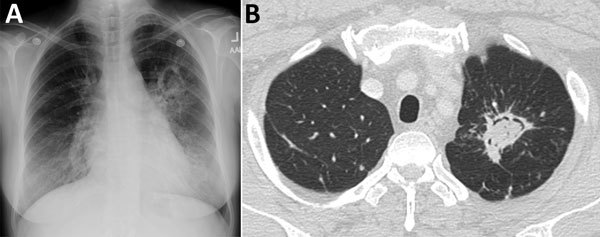
A) Chest radiograph showing aspergilloma (fungal ball) in the left upper lung lobe. B) Axial computed tomography image shows increased density in an irregular left apical cavity, a sequela of pulmonary tuberculosis, consistent with an aspergilloma.

The differential diagnosis for a fungal ball in the lung is limited: echinoccocal cyst, necrotizing bronchogenic carcinoma, or acute or subacute invasive fungal infection. Similar-appearing conditions are lung abscess, Rasmussen aneurysm in a tuberculous cavity, cavitating hematoma, or lung infarct (*34*). If single and in a localized area of lung, with few or no symptoms, a simple aspergilloma is the most precise diagnosis (Figure 1) and can be resected or observed without antifungal treatment. Although presence of a fungal ball is highly suggestive of an aspergilloma as a manifestation of CPA, microbiological confirmation is required for a definitive diagnosis.

Occasionally, a fluid level is visible in cavities. Few of these cavities have been sampled with aspiration and microbiological analysis, but when done, cultures are usually positive for *A. fumigatus* or, less commonly, *Staphylococcus aureus*, *Pseudomonas aeruginosa*, and other pathogens. Therefore, a fluid level in a cavity is entirely consistent with CPA but may also represent co-infection (*35*).

#### Cavitation

**Figure 2:**
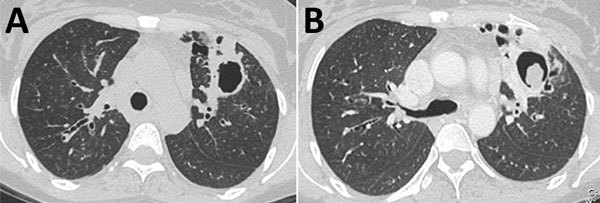
Computed tomography images of a patient with chronic pulmonary aspergillosis. A) Left upper lung lobe thick-walled cavity, showing associated pleural thickening. B) Same patient several months later, demonstrating progression of cavitation with increased pericavitary consolidation and formation of a fungal ball within the cavity. Aspergilloma formation is a late feature of chronic pulmonary aspergillosis.

The cardinal feature of CPA is the presence of >1 cavities. The cavities may be small or large; may have thick or, less commonly, thin walls; and usually abut the pleura (*33**,**36*). The cavities expand and may coalesce during progression of infection. In patients with extensive bullous emphysema, inflammation around a bulla may resemble cavitation. The differential diagnosis of chronic cavitary lesions includes mycobacterial infection, endemic fungal infection, and malignancy (*37*). The cavities seen with CPA are often located in the upper lung zone and may mimic TB; in patients with NTM infection and endemic fungal diseases, the cavities may be located in any lung zone. A distinguishing feature of CPA is slow progression of findings over months or years, whereas active pulmonary TB infection progresses faster (*34**,**38*) (Figure 2). Patients with NTM infection may also have cavitary lesions; however, lesions are more common among older white men with underlying lung disease (*39*). Patients with residual coccidioidal or other infectious cavities are usually asymptomatic unless these conditions are complicated by aspergilloma or superinfected (*40*). Cavitary bronchogenic carcinoma is usually associated with adenopathy and often pleural effusion.

#### Pleural Thickening

In the pre-CT era, pleural thickening was regarded as a sign of aspergilloma; indeed, pleural thickening is common in patients with CPA (*34**,**38*). For many patients, pleural thickening seen on CT images usually consists of 2 components: fibrosis of the pleura overlying a cavity or area of consolidation and indrawing of extrapleural fat (typically seen in chronic inflammatory processes of the lung). This subtle distinction cannot be made readily from a chest radiograph. Pleural thickening should be regarded as a common feature of CPA and useful for diagnosis. Furthermore, pleural thickening is a specific feature of CPA, rarely seen in patients with TB or chronic coccidioidal or other fungal cavities (*40**,**41*). Tuberculous empyema is usually exhibited as a large pleural effusion (*42*) and is commonly accompanied by interlobular septal thickening and micronodules (*43*). Cavitating bronchogenic carcinoma may invade the chest wall causing bone destruction or may cause diffuse pleural involvement; however, these manifestations are distinct from the focal pleural thickening associated with CPA.

#### Pericavitary Infiltration

The inflammatory changes seen adjacent to cavities in patients with CPA are often marked, reflecting inflammation but not hyphal invasion. These areas may merge with localized areas of fibrosis, pleural thickening, or both but are usually obvious on plain chest radiographs (*36*); they indicate active CPA and are a clear indication for therapy (*44*). Such pericavitary infiltrates, unless very extensive, are uncommon in reactivation of TB and NTM disease. Pericavitary consolidation may be seen in patients with chronic fibrocavitary coccidioidomycosis (*40*).

#### CT Image Features

**Figure 3:**
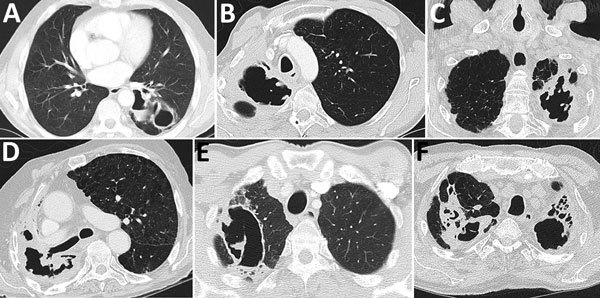
Computed tomography images showing early features of fungal ball formation in pulmonary cavities. A) Two left lower lung lobe posterior thick-walled cavities, 1 with a fluid level. B) Two right apical cavities, the larger with an irregular interior wall, most consistent with fungal growth. C) Left apex replaced by an irregular thick-walled cavity with multiple areas of fungal growth on the interior surface of the cavity. D) Substantial volume loss in the right upper lobe with replacement by a small anterior cavity and larger crescent-shaped cavity with both pleural thickening and fat indrawing along the pleural surface posteriorly. The cavity shows marked irregularity consistent with fungal growth. E) A right upper lobe thin-walled cavity containing 2 areas of fungal growth, 1 of which has detached from the wall as a thick mat of mycelial growth with a larger lump in the cavity interior. F) Multiple cavities in both upper lobes, with wall irregularity in the left upper lobe cavity consistent with surface fugal growth. The right upper lobe cavity shows pleural thickening and indrawing of fat posteriorly.

One radiologic aspect of CPA not characterized on a chest radiograph is the interior of a cavity. *Aspergillus* grows inside the cavity along the wall, resulting in an irregular appearance of the inner border seen on CT images. In addition, the cavity may contain linear opacities representing mats of fungal growth that have detached from the cavity wall (*38*). These growths often merge to form sponge-like densities, which can be described as a fungal ball containing air (*45*). These structures may detach from the cavity wall and may be mobile. For purposes of the definitions outlined in this article, all these characteristic features are deemed to be equivalent to a fungal ball (Figure 3). With antifungal therapy, they resolve more readily than an aspergilloma.

One feature for which CT is much more sensitive than chest radiography is the *Aspergillus* nodule, particularly when the nodule is small (*46*). These nodules may have a maximum diameter of 5–50 mm, may be single or multiple, and may be solid or have central cavitation. Larger nodules (>3 cm) are more accurately described as masses, which may also be attributable to *Aspergillus* infection. The differential diagnosis of the *Aspergillus* nodule is broad and includes carcinoma (primary or secondary), coccidioidomycosis, cryptococcosis, NTM infection, and others.

Establishing a definitive diagnosis of a nodule usually requires resection or biopsy, especially because many patients do not have elevated IgG against *Aspergillus* or positive sputum cultures (*47**,**48*). Because these diagnostic procedures are not available in many medical centers in lower- and middle-income countries, we elected to consider these separately and not include them in our operational definition.

### Culture-Based and Non–Culture-Based Evidence of Aspergillosis

The most reliable diagnostic test for CPA is a positive *Aspergillus* antibody test result, indicative of an immune response to *Aspergillus*. The second most reliable diagnostic marker for CPA is detection of *Aspergillus* in the airways by use of culture, antigen, and/or nucleic acid amplification (PCR).

#### Antibodies against *Aspergillus*

Elevated circulating levels of *Aspergillus* antibodies are present in >95% but <100% of patients (*49**–**51*). Most commercial assays detect *A. fumigatus* IgG; however, CPA is occasionally caused by other *Aspergillus* species, resulting in false-negative results. In addition, some patients with CPA are subtly immunocompromised, as has been documented with nonprotective pneumococcal or *Haemophilus* antibodies (*52*); low circulating CD4 (T helper), CD19 (B cell), or CD56 (natural killer) cell counts (*53*); and poor production of gamma interferon interleukin 17A, interleukin 12, or both (*54*). Such patients may not mount a detectable IgG response. Many patients have detectable *A. fumigatus* IgE and elevated total IgE in the absence of any other features of allergic *Aspergillus* disease (*21*).

Serologic testing for *Aspergillus* relies mainly on the detection of IgG and of precipitating antibodies (known as precipitins), which may be IgG or IgM. Precipitin detection requires immunodiffusion and electrophoresis migration methods, which lack standardization and are too laborious and time-consuming for resource-constrained settings. Consequently, we focused on commercially available enzyme immunoassay kits that detect IgG. Up to 2015, there were few direct comparisons of *A. fumigatus* serology (*55*), but more recently, several have been published (Table 2). Crucial to these comparisons is defining the cutoff values for each assay. The control groups of patients used for such comparisons have included healthy persons or those with pulmonary disease without aspergillosis. Few data on antibody titer associations with age, HIV status, or ethnicity of controls are available. New lateral flow devices for *Aspergillus* antibody detection are in the final stages of development and, if their performance is good, could greatly facilitate diagnosis. The consensus of the expert panel was that any *Aspergillus* antibody test performance had to be at least 90% sensitive and 85% specific.

**Table 2:** Performance of commercially available *Aspergillus* diagnostic serology tests for CPA*

Reference	Study population	Assay†	Cutoff	Sensitivity, %	Specificity, %	ROC/AUC (95% CI)
(*56*)	28 CPA patients	DD, Microgen	—	89.3	ND	ND
		Bio-Rad galactomannan	GM index >0.5	50	ND	ND
(*57*)	51 CPA patients and 341 controls‡	Bio-Rad Platelia *Aspergillus* IgG	10 AU/ml	90.2	89.6	ND
		Serion/Virion ELISA classic *Aspergillus* IgG	70 AU/mL	88.8	84.4	ND
(*25*)	49 simple aspergilloma patients	IBL culture filtrate ELISA	ND	99	ND	ND
		Bio-Rad galactomannan	GM index >0.5	23	ND	ND
(*58*)	116 CPA patients	Bio-Rad Platelia *Aspergillus* IgG	10 AU/mL	86	ND	ND
		ThermoFisher Scientific ImmunoCAP	40 mg/L	85	ND	ND
		DD		56	ND	ND
(*59*)	168 CPA patients	Bio-Rad galactomannan	GM index >0.5	23	ND	0.538 (0.496–0.580)
		DD	ND	98	ND	ND
(*60*)	89 CPA patients, 10 aspergilloma patients, 212 blood healthy donors	*Aspergillus* LDBio Western blot IgG kit	ND	CPA, 91.0; aspergilloma, 90.0	ND	ND
(*49*)	241 CPA patients, 100 blood donors from Uganda	Dynamiker	65 AU/mL	77	97	0.918 (0.89–0.946)
		Omega (Genesis) *Aspergillus* IgG ELISA kit	20 AU/mL	75	99	0.902 (0.871–0.933)
		Immulite Siemens	10 mg/L	96	98	0.991 (0.982–1)
		ThermoFisher Scientific Immunocap	20 mg/L	96	98	0.996 (0.992–1)
		Serion/Virion ELISA classic *Aspergillus* IgG	35 AU/mL	90	98	0.973 (0.96–0.987)
		Precipitins (Microgen)	ND	59	100	ND
(*50*)	17 simple aspergilloma patients, 62 CPA patients, 25 CNPA patients, 205 controls§	Bordier	OD>1	Simple aspergilloma, 95.6; CPA, 97.4; CNPA: 100	90.3	0.997 (0.962–0.991)
		Bio-Rad Platelia *Aspergillus* IgG	10 AU/mL	Simple aspergilloma, 95.6; CPA, 97.4; CNPA, 100	91.3	0.951 (0.928–0.974)
		Serion/Virion ELISA classic *Aspergillus* IgG	70 AU/mL	Simple aspergilloma, 78.3; CPA, 82.0; CNPA, 82.9	81.5	0.897 (0.863–0.931)
(*51*)	51 possible CPA patients, 96 proven CPA patients, 122 controls¶	ThermoFisher Scientific ImmunoCAP	50 mg/L	Possible CPA, 39.2; Proven CPA, 97.9	ND	0.94 (0.912–0.972)
(*61*)	241 CPA patients, 152 healthy controls from the Netherlands	Siemens Immulite	25 mg/L	92.9	99.3	0.948 (0.921–0.975)
	241 CPA patients, 141 healthy controls from Belgium	ThermoFisher Scientific ImmunoCAP	50 mg/L	83.8	95.6	0.956 (0.937–0.974)
	241 CPA patients, 222 healthy controls from France	Serion	50 U/mL	84.2	91	0.944 (0.925–0.964)
	118 CPA patients, 222 healthy controls from France	Bio-Rad	1.5 AU/mL	93.2	98.2	0.955 (0.922–0.988)
(*62*)	241 CPA patients, 299 healthy controls from Uganda	Siemens Immulite	15 mg/L	94.6	98	0.984 (0.972–0.997)
	241 CPA patients, 398 patients with treated TB from Uganda	Siemens Immulite	15 mg/L	94.6	94.5	0.972 (0.959–0.985)
	241 CPA patients, 234 patients with treated TB, radiologically screened for CPA from Uganda	Siemens Immulite	25 mg/L	92.9	98.7	0.979 (0.967–0.992)

*AU, absorbance units; CPA, chronic pulmonary aspergillosis; CNPA, chronic necrotizing pulmonary aspergillosis; DD, double diffusion (precipitins); GM, galactomannan; ROC/AUC, receiver operating characteristic/area under the curve; OD, optical density;TB, tuberculosis; ND, not determined (did not test enough control serum to assess). †Bio-Rad, Marnes-la-Coquette, France; Bordier, Crissier, Switzerland; DD, in-house test (*58*); Dynamiker, Tianjin, China; IBL, Hamburg, Germany; LDBio Diagnostics, Lyon, France; Microgen, Camberley, UK; Omega, Alva, Scotland, UK; Serion/Virion, Würzburg, Germany; Siemens, Camberley, UK; ThermoFisher, Uppsala, Switzerland. ‡Population included 26 patients with *Aspergillus* bronchial colonization, 44 patients with 1 positive *Aspergillus* culture considered as colonization, 49 patients with negative microbiological results, and 222 pregnant women. §Control groups comprised 14 patients colonized with *Aspergillus* and 191 patients with respiratory symptoms. ¶Possible CPA, *Aspergillus* precipitin negative and a persistently elevated inflammation marker; proven CPA, *Aspergillus* precipitin positive and a persistently elevated inflammation marker; control, other chronic respiratory disease (any *Aspergillus* precipitin and temporary elevated inflammation marker).

An elevated level of *Aspergillus* IgG is consistent with several conditions, including *Aspergillus* rhinosinusitis, allergic bronchopulmonary aspergillosis, *Aspergillus* bronchitis (notably in cystic fibrosis and bronchiectasis), subacute invasive aspergillosis, recovery from invasive aspergillosis, and community-acquired *Aspergillus* pneumonia. Because an elevated level of IgG is highly sensitive but not specific for CPA, the diagnosis of CPA requires the presence of compatible symptoms and radiologic abnormalities.

#### Respiratory Tract Microscopy and Culture

Microscopy of sputum may show hyphae morphologically consistent with *Aspergillus* spp. If present, this finding is most consistent with CPA or *Aspergillus* tracheobronchitis (*63*). Despite the substantial amount of *Aspergillus* spp. in the cavities of patients with CPA, culture positivity from sputum samples is lower than expected (41%–81%) and is probably biased toward culture-positive cases (*21**,**22**,**64**–**67*). One reason for this lower sensitivity is the inoculation of culture plates with very small volumes of sputum (*65**,**68*) as is done for bacterial culture. Negative cultures might reflect an inability of the fungus to adapt to in vitro conditions, despite the apparent ease with which environmental contamination occurs in the laboratory.

The merits of positive culture results are substantial, notably for identifying the *Aspergillus* species causing infection and enabling susceptibility testing to be performed. False-positive cultures do occur as a result of laboratory contamination. Although most CPA cases are caused by *A. fumigatus* complex (probably sensu stricto), *A. niger* complex and *A. flavus* complex and rare cases caused by unusual pathogenic species are reported. In countries such as India, where *A. flavus* infection is much more common, it is not clear what proportion of cases are attributable to non–*A.* *fumigatus* species. Most triazole-resistant *A. fumigatus* isolated from patients with CPA has arisen while the patient was receiving therapy and probably result from large fungal loads or low drug exposure (low dose, drug interactions, poor bioavailability) (*69*). Isolates may be resistant or have intermediate susceptibility to 1–4 triazoles.

#### Aspergillus Antigen and β-1,3-D-glucan 

Galactomannan is a detectable carbohydrate antigen produced by the growth of *Aspergillus* spp. Galactomannan detection is useful for the diagnosis of invasive aspergillosis because it is often detectable in serum and bronchoalveolar lavage (BAL) fluid. Because tissue invasion does not occur in patients with CPA, galactomannan is not usually detectable in serum and is usually detectable in BAL fluid only (*59**,**70*). This finding is of limited utility in lower- and middle-income countries because fiberoptic bronchoscopy is infrequently done. Galactomannan is detectable in sputum or tracheal secretions/aspirates, but the cutoff for positivity is not established (*71*).

A new simple lateral flow assay for different protein antigens specific to *A. fumigatus* has been commercialized, but no data are available on sputum detection or its utility for patients with CPA. This assay is unlikely to be useful in serum because of antibody masking but may be useful in BAL fluid (*72**,**73*). β-1,3-D-glucan is released by *Aspergillus* spp. (and many other fungi) during infection but is less specific and probably not more sensitive than serum galactomannan in patients with CPA.

#### Molecular Detection of *Aspergillus*

For patients with CPA, PCR detection of *Aspergillus* spp. in sputum is significantly more sensitive than culture (*63*). However, the quality and possibly quantity of the sputum sample influence PCR performance (*71*). Furthermore, studies have shown that the sensitivity of PCR for detection of *Aspergillus* is ≈80%. Depending on the cutoff value used, low specificity may also be an issue. Molecular assays for *Aspergillus* spp. are not routinely available in most medical centers worldwide, especially in lower- and middle-income countries.

### Consensus Definition and Proposed Algorithm

**Figure 4:**
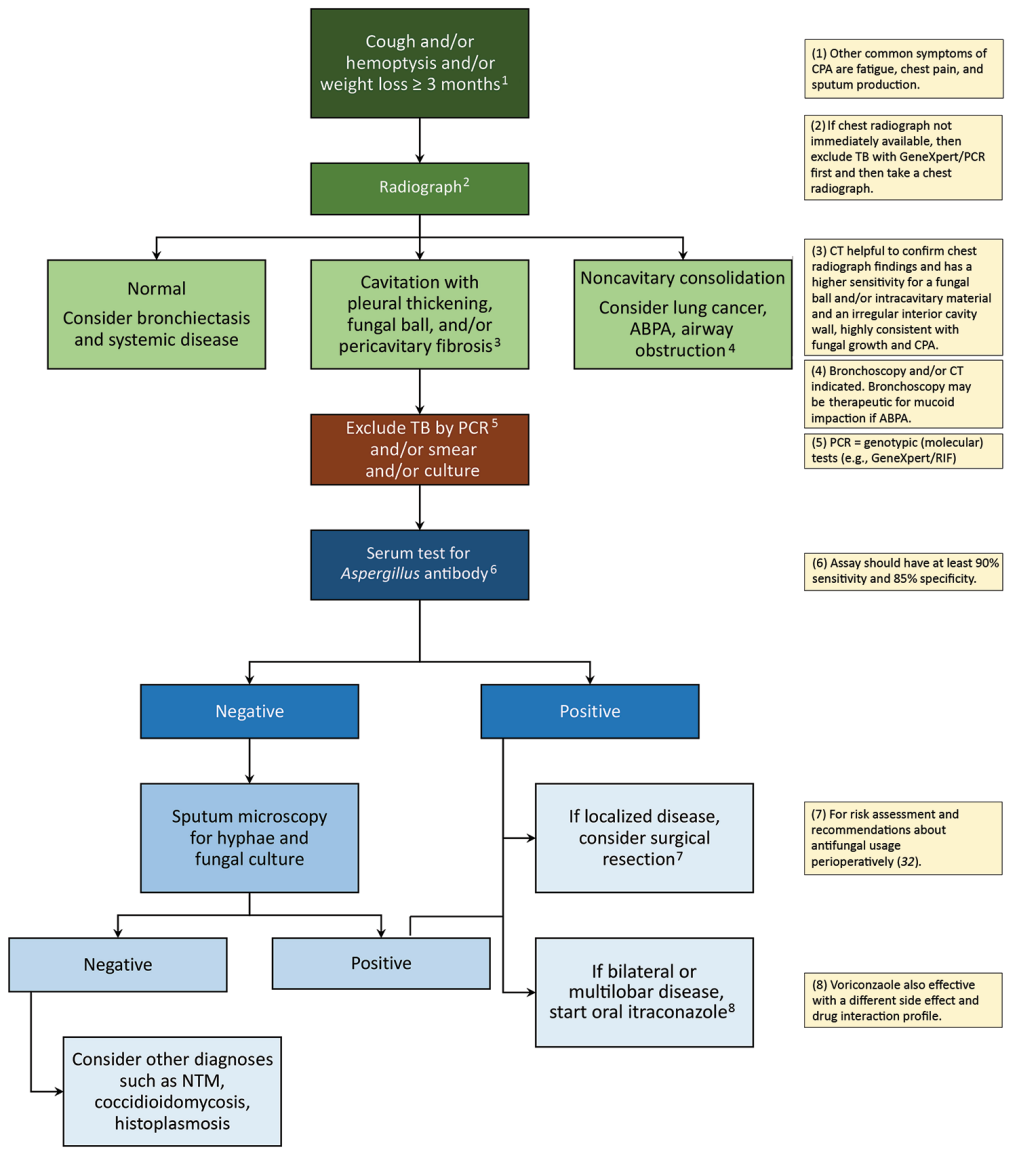
Diagnostic algorithm incorporating the chest radiographic appearance and results of rapid TB investigations with the case definition of CPA. ABPA, allergic bronchopulmonary aspergillosis; CPA, chronic pulmonary aspergillosis; CT, computed tomography; NTM, nontuberculous mycobacteria; TB, tuberculosis. GeneXpert, http://www.cepheid.com/us/cepheid-solutions/systems/genexpert-systems/genexpert-iv

The definition of CPA recommended for use in resource-constrained settings in shown in Table 3. In a patient who has had symptoms consistent with chronic *Aspergillus* infection for >3 months, a chest radiograph should be obtained (Figure 4). If a chest radiograph is not possible, then pulmonary TB should be excluded by examination of respiratory specimens. The chest radiograph appearance determines the next course of action. If the chest radiograph is not remarkable, the signs and symptoms may be caused by bronchiectasis or systemic disease. If the chest radiograph shows consolidation without cavitation, then other diagnoses such as lung malignancy, airway obstruction, or endemic fungal infections should be considered. If the chest radiograph shows cavitation, then work-up for possible pulmonary TB should be initiated according to national practice and standards. A positive result for pulmonary TB should lead to appropriate treatment. If the pulmonary TB results are negative, and especially if the radiographic findings include pleural thickening, fungal ball, or pericavitary fibrosis or infiltrates, serum should be tested for *Aspergillus* IgG (Figure 4). If the *Aspergillus* IgG test result is negative (or unavailable), then sputum microscopy for hyphae or fungal culture should be performed; yield is higher when multiple specimens are tested. If the sputum microscopy for hyphae or fungal culture is negative, then other diagnoses, such as atypical mycobacterial infection or endemic fungal infection, should be considered. If sputum microscopy for hyphae or culture is positive, or if a serum *Aspergillus* antibody test result is positive, then CPA is confirmed and treatment with itraconazole or voriconazole is advised (*19*).

**Table 3:** Final consensus definition of CPA in resource-constrained settings, determined by Global Action Fund for Fungal Infections international expert panel*

Required criteria†	Details
Symptoms for >3 mo	Hemoptysis and/or persistent cough and/or weight loss; Other symptoms are common, but not requred, notably fatigue, chest pain, dyspnea, and sputum production
Radiologic features	Progressive cavitation on chest imaging and/or intracavitary fungal ball and/or pleural thickening or pericavitary fibrosis or infiltrates all adjacent to cavities
Microbiological evidence of *Aspergillus* infection	Positive *Aspergillus*-specific IgG and/or sputum microscopy results showing hyphae consistent with *Aspergillus* and/or *Aspergillus* growth on >2 sputum or other respiratory samples
Mycobacterial infection ruled out with smear, GeneXpert, and/or mycobacterial culture‡	It is possible for mycobacterial infection and CPA to be present concurrently, but this diagnosis requires characteristic radiological findings on CT scan that are not present with pulmonary TB including pleural thickening, a fungal ball or other intracavitary material, or marked pericavitary infiltrates in addition to a positive *Aspergillus* IgG antibody test

*CPA, chronic pulmonary aspergillosis; CT, computed tomography; TB, tuberculosis. †All 4 criteria are required. ‡GeneXpert (http://www.cepheid.com/us/cepheid-solutions/systems/genexpert-systems/genexpert-iv).

## Conclusions

We present recommendations for diagnosing CPA, adapted for clinical use and public health surveillance in resource-constrained settings. The adapted definition relies on a combination of signs and symptoms, chest radiograph features, and serologic evidence of *Aspergillus* infection; it is applicable to almost all patient groups in which CPA occurs. Prospective evaluation of the proposed definition and associated algorithms will be valuable in the contexts of different geographic and HIV and pulmonary TB burdens (*74*). We believe that the proposed operational definition of CPA in lower- and middle-income countries will facilitate advancements in research and practice (clinical, laboratory, radiologic, and public health) and policy (health services and public health) in these countries and globally in resource-constrained settings.
